# Tolerance Induction to Cytoplasmic β-Galactosidase by Hepatic AAV Gene Transfer — Implications for Antigen Presentation and Immunotoxicity

**DOI:** 10.1371/journal.pone.0006376

**Published:** 2009-08-04

**Authors:** Ashley T. Martino, Sushrusha Nayak, Brad E. Hoffman, Mario Cooper, Gongxian Liao, David M. Markusic, Barry J. Byrne, Cox Terhorst, Roland W. Herzog

**Affiliations:** 1 Department of Pediatrics, University of Florida, Gainesville, Florida, United States of America; 2 Beth Israel Deaconess Medical Center, Harvard Medical School, Boston, Massachusetts, United States of America; University of Michigan, United States of America

## Abstract

**Background:**

Hepatic gene transfer, in particular using adeno-associated viral (AAV) vectors, has been shown to induce immune tolerance to several protein antigens. This approach has been exploited in animal models of inherited protein deficiency for systemic delivery of therapeutic proteins. Adequate levels of transgene expression in hepatocytes induce a suppressive T cell response, thereby promoting immune tolerance. This study addresses the question of whether AAV gene transfer can induce tolerance to a cytoplasmic protein.

**Major Findings:**

AAV-2 vector-mediated hepatic gene transfer for expression of cytoplasmic β-galactosidase (β-gal) was performed in immune competent mice, followed by a secondary β-gal gene transfer with E1/E3-deleted adenoviral Ad-LacZ vector to provoke a severe immunotoxic response. Transgene expression from the AAV-2 vector in ∼2% of hepatocytes almost completely protected from inflammatory T cell responses against β-gal, eliminated antibody formation, and significantly reduced adenovirus-induced hepatotoxicity. Consequently, ∼10% of hepatocytes continued to express β-gal 45 days after secondary Ad-LacZ gene transfer, a time point when control mice had lost all Ad-LacZ derived expression. Suppression of inflammatory T cell infiltration in the liver and liver damage was linked to specific transgene expression and was not seen for secondary gene transfer with Ad-GFP. A combination of adoptive transfer studies and flow cytometric analyses demonstrated induction of Treg that actively suppressed CD8^+^ T cell responses to β-gal and that was amplified in liver and spleen upon secondary Ad-LacZ gene transfer.

**Conclusions:**

These data demonstrate that tolerance induction by hepatic AAV gene transfer does not require systemic delivery of the transgene product and that expression of a cytoplasmic neo-antigen in few hepatocytes can induce Treg and provide long-term suppression of inflammatory responses and immunotoxicity.

## Introduction

In the mid 1990s, *in vivo* gene transfer with viral vectors was either inefficient for target cells that were not actively dividing (in the case of retroviral gene transfer) or resulted in robust but only transient gene expression because of cytotoxic T lymphocyte (CTL) responses (in the case of adenoviral vectors). For example, first generation adenoviral vectors carrying a LacZ reported gene yielded only transient expression (less than 1 month) of the β-galacosidase (β-gal) enzyme upon gene transfer to the liver or to skeletal muscle because of CTL responses to β-gal and to viral antigens [1]–[4]. However, in 1996–7, four laboratories reported sustained expression of β-gal in skeletal muscle fibers of immune competent animals using the same CMV enhancer/promoter driven expression cassette delivered by an adeno-associated viral (AAV) vector instead of adenovirus [5]–[8]. AAV gene transfer appeared much stealthier and did not activate β-gal specific CTLs, nor did this vector contain viral coding sequences. These findings sparked numerous investigations on recombinant AAV, making it one of the most popular gene therapy vectors, in particular for *in vivo* gene transfer [9]–[11]. AAV vectors are derived from a non-pathogenic member of the parvovirus family that is naturally replication deficient and is comprised of a 4.7-kb single-stranded DNA genome packaged into a viral capsid. Recent successes with AAV-mediated gene transfer include successful treatment of patients with Leber's Congenital Amaurosis, a rare form of inherited blindness, correction of sarcoglycan deficiency in skeletal muscle of dystrophic patients, and multi-year correction of hemophilia in canine models by a single hepatic administration [12], [13].

A major concern in treatment of genetic disease is that the therapeutic gene product, which is used to replace the endogenous, non-functional or entirely absent protein, represents a novel antigen to the immune system [14]–[19]. Therefore, adaptive immunity may cause formation of antibodies or CTL responses against this protein. The latter target the expressing cells, thereby eliminating gene corrected cells. In the case of AAV-LacZ gene transfer to skeletal muscle, subsequent studies showed that the β-gal antigen was hidden from the immune system via an ignorance mechanism, in part because of a lack of expression of the transgene in antigen presenting cells (APCs), attributed to low *in vivo* transduction efficiency of dendritic cells (DCs) and macrophages with the AAV vector, and in part because of the cytoplasmic localization of the gene product [4], [20].

A number of recent studies have demonstrated induction of immune tolerance to different protein antigens by hepatic gene transfer. Hepatocyte-derived antigen can induce a regulatory response, mediated by Treg, that actively suppresses humoral and cellular immune responses [21]–[23]. In this context, AAV vectors were found to be some of the most suitable vehicles for tolerogenic transgene expression. However, tolerance induction by hepatic AAV gene transfer has been predominantly, if not exclusively, described for secreted or exocytosed proteins, resulting in systemic delivery of the antigen for cross-correction of a deficiency in other cell types or tissues or for correction of a blood disorder. Although hepatic expression is crucial for tolerance induction, systemic expression following secretion from hepatocytes leads to antigen presentation in many extra-hepatic sites, which could represent an important contributing factor to tolerance induction [24]–[26]. We therefore asked the question of whether expression of a non-secreted cytoplasmic antigen from an AAV vector would induce immune tolerance.

We found that transgene expression in few hepatocytes (<3%) from an AAV vector resulted tolerance induction to cytoplasmic β-gal (rather than ignorance). Tolerized mice lacked antibody and CTL responses to β-gal and contained Treg capable of suppressing β-gal specific CD8^+^ T cells. Substantial reduction of immunotoxicity upon secondary gene transfer with an adenoviral vector expressing β-gal correlated with increases of FoxP3^+^ Treg frequencies.

## Materials and Methods

### Ethical statement

All animal work has been performed according to University of Florida IACUC regulations.

### Viral Vectors

AAV-LacZ vector (serotype 2) contained the β-gal cDNA under transcriptional control of the hepatocyte-specific Duck Hepatitis B Virus promoter [27]. This vector was produced by transient transfection in HEK-293 cells, purified, and titered as previously described. Vector doses are reported in vector genomes [vg; as determined by quantitative slot blot hybridization] [28]. E1/E3-deleted adenoviral vectors expressed green fluorescent protein (Ad-GFP) or β-gal (Ad-LacZ) from the CMV I.E. enhancer/promoter [19], [29]. These vectors were produced in HEK-293 and purified by CsCl gradient centrifugation using standard protocols. Viral doses are reported as viral particles [vp] as determined by absorbance measurement.

### Animal experiments

**Figure 1:**
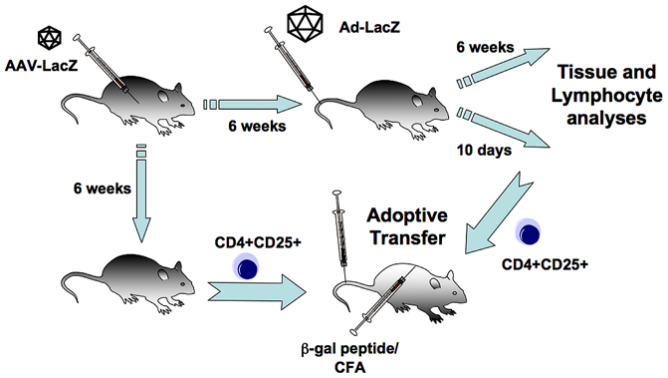
Flow chart of experimental design. First, AAV2-LacZ hepatic gene transfer was performed. A secondary I.V. delivery of Ad-LacZ (or Ad-GFP) was performed 6 weeks later or in naïve mice to provoke an immunotoxic response. Samples were taken and experiments were performed 10 and 45 days after Ad delivery. Splenic CD4^+^CD25^+^ T cells were adoptively transferred to naïve mice 6 weeks after AAV2-LacZ gene transfer or 10 days after secondary Ad-LacZ delivery. Recipient mice were immunized with a peptide encoding the dominant CD8^+^ T cell epitope for β-gal in this strain. All animals were C57BL/6 mice.

Mice used in this study were the C57BL/6 strain from Jackson Laboratories and FoxP3-IRES-EGFP knock-in mice on BALB/c background. Foxp3-IRES-EGFP mice co-express EGFP and the regulatory T cell-specific transcription factor Foxp3 as described by others [30]. AAV-LacZ vector was delivered at 2×10^11^ vg/mouse into the portal circulation by splenic capsule injection as published [18]. Ad-LacZ or Ad-GFP was delivered at 1×10^11^ particles by tail vein injection (IV) to naïve mice or mice that had received AAV-LacZ gene transfer 6 weeks earlier. Mice were sacrificed at two different times after Ad administration depending on the focus of the study: 10 days (immunotoxicity during acute inflammation) or 45 days (for post inflammation status) after adenoviral gene transfer (Fig. 1). Livers, spleens, and blood samples were collected for analysis. Additionally, in the D+45 groups of mice, animals were bled at Day 0, Day 14, and Day 28 to measure antibody titers against β-gal.

### Adoptive T cell transfer

Spleens from viral vector transduced C57BL/6 mice were harvested into 2-MLC media (DMEM, 2% heat-inactivated fetal calf serum, 1 mM sodium pyruvate, 10 mM HEPES, 0.1 mM non-essential amino acids, 10^−6^ M 2-mercaptoethanol, and antibiotics) at room temperature, homogenized, filtered through a 70-µm cell strainer, and centrifuged at 300 g for 10 min. Cells were subsequently incubated with ACK lysing buffer (BD Bioscience) for 5 min and washed twice with 2-MLC medium. Viable splenocytes were counted with a hemacytometer and trypan blue. The CD4^+^CD25^+^ T cell isolation kit (Miltenyi, Auburn, CA) was used to purify Tregs from bulk splenocytes by magnetic cell sorting as described [25]. Purified CD4^+^CD25^+^ cells were pooled for each experimental group of donor mice and delivered to naïve C57BL/6 mice at 1×10^6^ by tail vein injection (Fig. 1). Recipient mice were immunized with 5 µg of the DAPIYTNV (the β-gal dominate CD8^+^ T cell epitope in C57BL/6 mice [31]) emulsified in Complete Freund's Adjuvant (CFA) by 4 dorsal subcutaneous injections 24 hours after adoptive transfer. Mice were sacrificed 14 days after challenge to determine frequencies of IFN-γ T cells by ELISpot.

### Measurement of CD4^+^CD25^+^Foxp3^+^ T cell frequencies by flow cytometry

Splenocytes were isolated as described above. Nuclear Stain for transcription factor FoxP3 was performed with the BD Biosciences (San Jose, CA) kit. Briefly, splenocytes (1×10^6^ per mouse) were stained with fluorescent conjugated antibodies, including anti-mouse CD4 - pacific blue (channel 6), anti-mouse CD25 - PE (channel 2) and anti-mouse FoxP3 - Alexa Fluor® 647 (channel 4) according to BD Biosciences staining protocol. Frequencies of CD25^+^Foxp3^+^ cells among total CD4^+^ splenocytes were assessed by FACS analysis using LSR-II instrumentation from BD Biosciences (San Jose, CA) and the CELLQUEST software.

### ELISpot for IFN-γ secreting cells

Splenocytes were isolated 14 days after either IV challenge with Ad-LacZ or subdermal challenge with the CD8^+^ β-gal dominant epitope, and cultured for ELISpot analysis. ELISpot was performed as previously described [15]. A 96 well ELISpot culture plate (Millipore, Billerica, MA) was pretreated with an IFN-γ capture antibody (BD Biosciences, San Jose, CA) overnight at 37°C in 10% CO_2_ at 1∶60 dilution in 100 µl of culture media (DMEM media with 5% FBS and 1% pen/strep). After overnight incubation, the wells were blocked using PBS with 5% sucrose and 1% BSA for 2 hours at room temperature. Subsequently, 1×10^6^ splenocytes were added in triplicate and cultured in a final volume of 200 µl of media for 30 hours at 37°C in 10% CO_2_. The stimulation media contained 5 µg/ml of the CD8^+^ b-gal dominant epitope or 250 viral paricles per cell of heat-inactivated (1 hour at 60°C) Ad-F.IX. Positive control stimulation media contained 10 µg/ml of Staphylococcal Enterotoxin B (SEB) super antigen, and mock media was the culture media without additional antigen. After stimulation, the wells were rinsed, and a secondary detection IFN-γ antibody at 1∶60 dilution in 100 µl filtered PBS with 1% BSA was added overnight at 4°C. Detection utilized HRP streptavidin (BD Biosciences, San Jose, CA) at 1∶60 dilution in 100 µl PBS/10% FBS for 2 hours at room temperature, followed by color development with 100 µl of BCIP/NBT chromogen (BD Biosciences). IFN-γ producing cells were analyzed and counted with the CTL-ImmunoSpot® S5 UV analyzer (Cellular Technology, Shaker Heights, OH).

### β-galactosidase enzymatic staining in mouse liver sections

Liver samples were fixed (n = 4/group) for 2 hours in 4% paraformaldehyde, rinsed in PBS, and frozen in OCT compound (using a 90% isoproponal dry ice bath) and stored at −80°C until sectioned. OCT blocks were sectioned (8 µM) with a cryostat, fixed for 10 min with cold acetone (4°C), rinsed with PBS, and immersed in β-gal enzyme activity stain buffer (X-gal buffer) at 37°C for 8 hours. Slides were rinsed with PBS and later stained with hemotoxylin for nuclear detection in order to determine the percentage of hepatocytes that stained positive for β-gal activity. X-gal buffer consisted of PBS with 2 µM MgCl_2_, 30 mM K_3_Fe(CN)_6_, 30 mM K_4_Fe(CN)_6_, and 1 µg/ml x-gal. For each liver, 5 random fields of view (at a total magnification of 100X) from 3 different sections separated by 24 microns where captured by the Nikon Eclipse 80i microscope equipped with a Photometric CoolSnap camera. The mean percentage of hepatocytes producing β-gal from these images was calculated using Pro Image Software.

### H&E staining and scoring of hepatic inflammation

OCT blocks were sectioned (8 µM) with a cryostat, fixed for 10 minutes with 4°C acetone, rinsed with PBS and stained by H&E (hemotoxylin & eosine). Slides were reviewed by one observer, blinded to treatment group, using the following scale to score inflammation within the liver parenchyma and portal ducts: 0 = none; 1 = focal, mild lesions; 2 = multifocal, moderate lesions; 3 = multifocal, severe lesions; 4 = multifocal degeneration or necrosis.

### Immunofluorescence staining of mouse liver sections

Livers samples from each group (n = 4/group) were frozen in OCT compound and sectioned as described above. Sections on slides were fixed for 10 min with room temperature acetone, allowed to dry for 30 minutes (to permeabilize the cells), rinsed with PBS, and stained with immunoflourescently labeled antibodies against CD8 and β-gal. Staining was performed by blocking with PBS with 5% goat serum for 1 hr at room temperature in a humidity chamber. Rat anti-mouse CD8a (eBiosciences, San Diego, CA) and rabbit anti-β-gal (Invitrogen, Carlsbad, CA) were added at 1∶100 and 1∶1000 dilution, respectively, in PBS/1% goat serum and incubated in a humidity chamber at 4°C overnight. Fluorescent conjugated goat anti-rat IgG Alexa Fluor® 568 (Invitrogen, Carlsbad, CA) and goat anti-rabbit IgG Alexa Fluor® 488 (Invitrogen, Carlsbad, CA) were added at 1∶200 and 1∶1000, respectively, and incubated for 1 hr at room temperature in a humidity chamber. Slides were mounted with fluoromount media (Fisher Scientific), and staining was captured using a Nikon Eclipse 80i fluorescent microscope equipped with a Photometric CoolSnap camera. Five random fields of view at 200X from 3 different sections separated by 24 microns were used to determine the level of CD8^+^ and β-gal^+^ staining. The ratio of hepatocytes producing the transgene product and CD8^+^ cells was determined using Pro Image Software.

### Liver enzyme levels

Blood was collected and plasma isolated from mice at day 10 after Ad I.V. gene transfer to determine level of ALT (alanine aminotransferase) and AST (aspartate aminotransferase), which are indicators of liver damage. ALT and AST levels were measured by the clinical chemistry lab at the small animal hospital affiliated with the University of Florida Veterinary Program (Gainesville, FL).

### Antibody levels against β-gal

Blood was collected from AAV-LacZ only, Ad-LacZ only, and AAV-LacZ plus Ad-LacZ transduced mice at Day 0, Day 14, Day 28 and Day 45 after IV delivery of Ad-LacZ. IgG1 and IgG2a immunocapture assays to determine antibody titers against β-gal was performed as follows. ELISA plates were coated with 1 ng/µl of recombinant β-gal protein (Sigma, St Louis, MO) overnight at 4°C. In parallel, 2-fold serial dilutions of mouse IgG1 or IgG2a (Sigma) were used to coat wells for standard curve. Blocking was done with dilution buffer (PBS containing 5% BSA and 0.05% Tween20) for 1 hr at room temperature. Samples were added at 1∶20 for 2 hours at 37°C. Goat anti-mouse IgG1 or IgG2a HRP-conjugated secondary antibody (Southern Biotech, Birmingham, AL) was added at 1∶2000 in dilution buffer for 2 hours at 37°C. Detection was done using SIGMA*FAST™* OPD tablets (Sigma). Absorption (OD_450_) was measured using the Model 680 microplate reader (Bio-Rad, Hercules, CA).

### Statistics

Statistical comparisons between experimental groups were performed by two-tailed Student's *t* test. Values were considered to be statistically significant for P<0.05.

## Results

### Protection of β-gal expression in hepatocytes following AAV gene transfer

In previous studies, we have demonstrated induction of immune tolerance to coagulation factor IX (F.IX) by hepatic AAV gene transfer [22]. In this study, we chose β-gal as a model antigen for a cytoplasmic expressed protein. Gene transfer was performed in C57BL/6 mice for the following reasons. This strain mounts effective CD8^+^ T cell responses against β-gal expressing cells (such as hepatocytes or myofibers) upon adenoviral gene transfer; it is known that following AAV gene transfer to skeletal muscle, lacZ expression in myofibers of C57BL/6 mice persists because of ignorance but is eliminated by a CTL response upon secondary gene transfer with Ad-LacZ; the immunodominant CD8^+^ T cell epitope for β-gal is known [3], [4], [31]–[34]. An outline of our experimental approach is described in Fig. 1.

**Figure 2:**
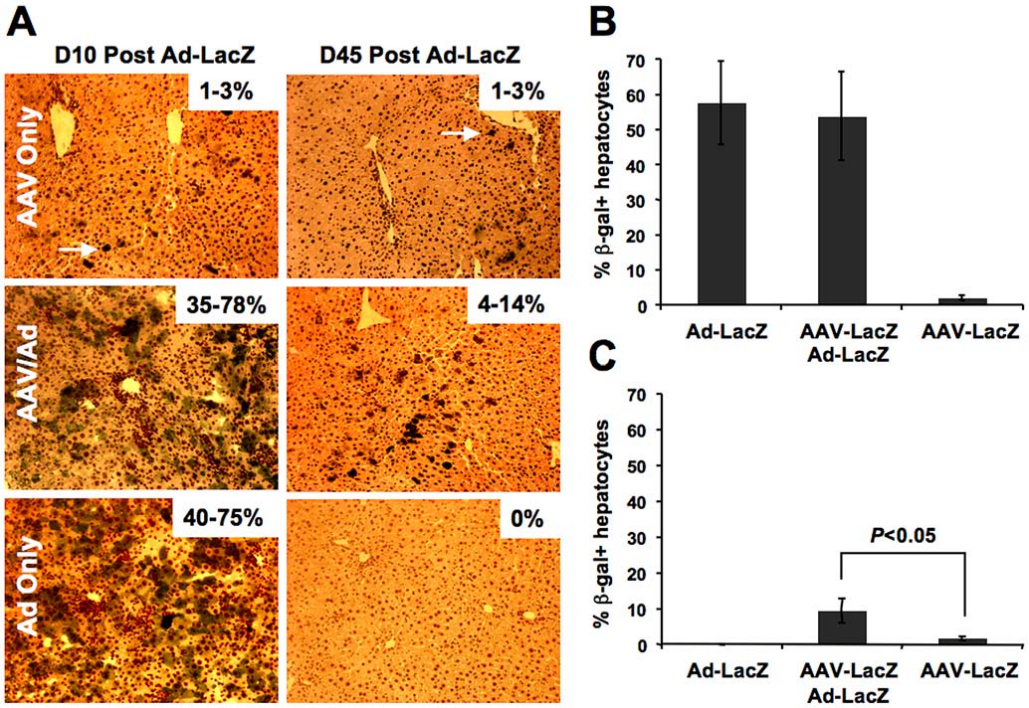
Expression of β-gal in viral vector transduced livers. Sections from C57BL/6 mice (3 per animal; separated by 24 microns) were stained for β-gal enzyme activity and counterstained with hemotoxylin nuclear stain. A. Representative images of livers from AAV-LacZ transduced mice (6 weeks or 12 weeks after gene transfer); AAV-LacZ/Ad-LacZ transduced mice (10 days or 45 days after Ad-LacZ administration, which occurred 6 weeks after the initial AAV-LacZ gene transfer); and Ad-LacZ only transduced mice (10 and 45 days after gene transfer). Arrows point toward examples of β-gal^+^ hepatocytes in AAV-LacZ only transduced livers. Original magnification: 100x. B-C. Percentages of β-gal expressing hepatocytes at day10 (B) or day 45 (C) after Ad-LacZ gene transfer. Data are average±SD for n = 4 mice/experimental group.

First, the capacity for hepatic AAV gene transfer to develop tolerance to a cytoplasmic transgene product and to protect against immunotoxic responses was explored. AAV-LacZ transduced livers showed a low level of transgene expression in 1–3% of hepatocytes when analyzed 45 days after gene transfer (Fig. 2). Ad-LacZ gene transfer resulted in transient high-level β-gal activity (40–75% of hepatocytes) at 10 days, which, as expected, declined to undetectable by 45 days (Fig. 2A-C). In contrast, livers initially transduced with AAV-LacZ had β-gal expression in 35–78% of hepatocytes and continued to express in a range of 4%–14% when analyzed 45 days after secondary gene transfer with Ad-LacZ (which was performed 45 days after AAV-LacZ transduction). These results demonstrate that a portion of the additional β-gal expression introduced by the more effective but highly immunogenic Ad-LacZ vector remained protected. This is in contrast to findings by others on muscle gene transfer, showing that secondary Ad-LacZ gene transfer eliminates previously AAV-LacZ transduced skeletal muscle fibers [4].

### Reduction of Ad-LacZ induced immunotoxicity by prior transduction with AAV-LacZ

**Figure 3 pone-0006376-g003:**
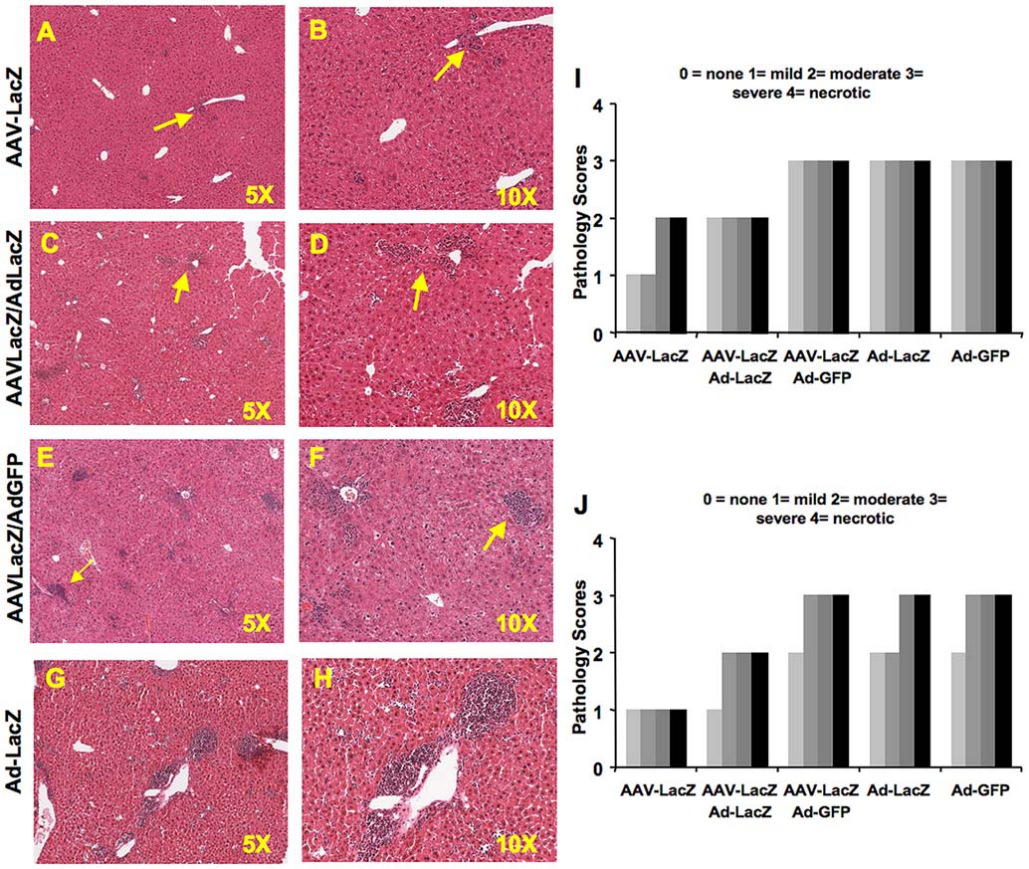
Liver inflammation. Liver sections were stained with H&E to determine levels of inflammation in the parenchyma and portal ducts at 10 days after and Ad-LacZ or Ad-GFP gene delivery to naïve mice or mice that had received AAV-LacZ gene transfer 6 weeks earlier. AAV-LacZ only transduced mice are shown for comparison (n = 4 per experimental group). A-H. Representative images at original magnification of 50x and 100x illustrate degrees of inflammation from AAV-LacZ, AAV-LacZ/Ad-LacZ, AAV-LacZ/Ad-GFP and Ad-LacZ only groups. A trained pathologist scored the liver inflammation in parenchyma (I) and portal ducts (J) in a blinded fashion to accurately determine the level of inflammation in the groups. Bars represent scores for livers of individual animals.

Since we observed partial protection of Ad-LacZ transduced hepatocytes, we decided to assess the level of hepatotoxicity induced by Ad-LacZ in naïve control and AAV-LacZ pre-treated mice (n = 4 per group) by assessment of liver inflammation and measuring the systemic levels of liver enzymes as indicators of liver damage. In mice that had received AAV-LacZ only, there were 2 cases of mild inflammation and 2 cases of moderate inflammation in the parenchyma, and 4/4 animals had only mild inflammation in the portal ducts (Fig. 3A, B, I, J). All (4/4) Ad-LacZ only transduced livers presented with severe inflammation in the parenchyma and 2 cases of moderate and 2 cases of severe inflammation in the portal ducts (Fig. 3G, H, I, J). Additional control groups transduced with Ad-GFP vector only or receiving AAV-LacZ followed by Ad-GFP showed similar levels of severe inflammation (Fig. 3E, F, I, J). The AAV-LacZ/Ad-LacZ group, however, showed 4 of 4 livers with moderate inflammation in the parenchyma and 3 cases of moderate and 1 case of mild inflammation in the portal ducts (Fig. 3 C, D, I, J). In summary, these results reflected a level of inflammation that was intermediate between AAV-LacZ only and Ad-LacZ only treated mice.

**Figure 4:**
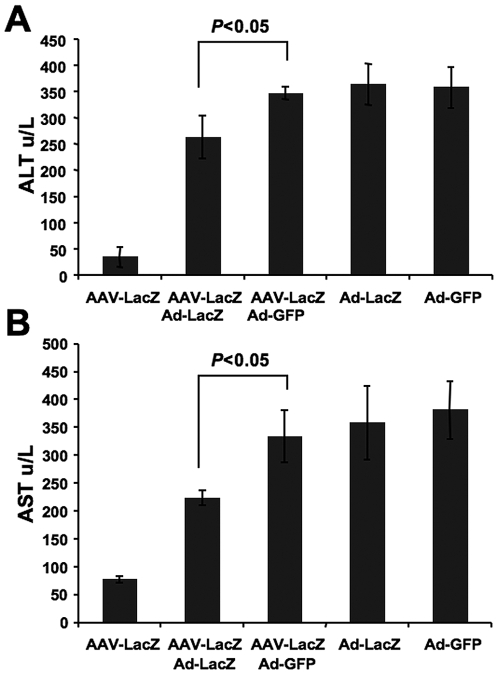
Systemic levels of liver enzymes as indicators of liver damage. Systemic levels of AST (aspartate aminotransferase) and ALT (alanine aminotransferase) liver enzyme levels were measured from plasma for all experimental groups of C57BL/6 mice 10 days after Ad-LacZ or Ad-GFP delivery (i.e. during the acute inflammatory response in the liver) and compared to data from AAV-LacZ transduced mice that did not receive Ad vectors. Data are average±SD for n = 4 mice/experimental group.

Systemic ALT and AST levels, indicators of liver damage, were within normal range (40 U/L and 70 U/L, respectively) in the AAV-LacZ only group (Fig. 4). In the AAV2-LacZ/Ad-LacZ group, ALT levels were elevated to 250 u/L and AST levels elevated to 225 U/L, but were significantly reduced compared to the AAV-LacZ/Ad-GFP, Ad-LacZ only, and Ad-GFP only groups, which were 350 u/L (ALT) and 325–375 U/L (Fig. 4). These measurements were performed on plasma samples obtained 10 days after adenoviral gene transfer.

### Severity of CD8^+^ cellular infiltrates in liver parenchyma

**Figure 5:**
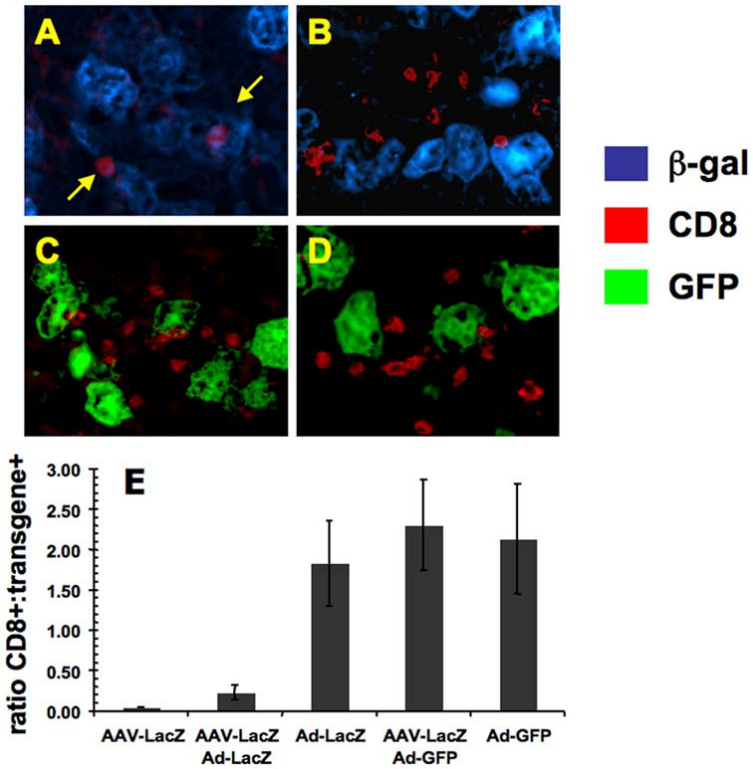
CD8^+^ cellular infiltrates in adenoviral transduced livers. Liver sections from naïve or AAV-LacZ pre-treated C57BL/6 mice 10 days after Ad-LacZ or Ad-GFP gene transfer. CD8^+^ cells were stained red with AlexaFluor® 568 while β-gal^+^ cells were stained green with AlexaFluor® 488. GFP^+^ cells were green by native fluorescence. A blue-pseudo color after image capture was used to show β-gal^+^ cells from A. AAV-LacZ/Ad-LacZ and B. Ad-LacZ livers, and the native green fluorescence was used to show GFP^+^ cells from C. AAV-LacZ/Ad-GFP and D. Ad-GFP livers. E. The ratio of CD8^+^ staining surrounding either β-gal or GFP producing hepatocytes was determined by capturing the images at 200X and analyzing with Image Pro software. Data are average±SD for n = 4 mice/experimental group. Arrows in A. point toward CD8^+^ cells.

It is known that administration of a first generation adenoviral vector causes CD8^+^ T cell responses against transgene product and viral antigens encoded by the vector backbone. Co-staining of CD8^+^ cells and transgene expressing cells (either β-gal or GFP) was used to determine levels of CD8^+^ cellular infiltrate at the sight of transgene production at 10 days after Ad-LacZ or Ad-GFP vector delivery. Immunofluorescent detection showed close to a 2∶1 ratio of CD8^+^ cells to either β-gal^+^ or GFP^+^ hepatocytes in livers of Ad-LacZ, Ad-GFP, or AAV-LacZ/Ad-GFP transduced mice, while this ratio was 0.2∶1 in the AAV-LacZ/Ad-LacZ group. Therefore, this difference represents a 10-fold decrease in the CD8^+^ cellular infiltrate in the AAV-LacZ/Ad-LacZ mice (Fig. 5). Taken together, the results shown in Figs. 2–4 demonstrate that tolerance induction to β-gal by AAV gene transfer substantially reduces transgene product-directed immunotoxicity and that the reduction of adenoviral gene transfer-induced liver toxicity and hepatic CD8^+^ T cell infiltrate is linked to β-gal transgene expression. Toxicity and T cell responses to the liver remain high in secondary gene transfer with an Ad vector expressing a different transgene product.

### Humoral response to β-gal

**Figure 6:**
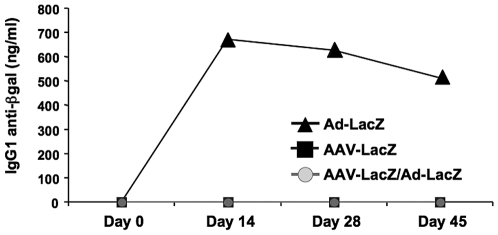
Antibody formation against β-gal. Graphed are β-gal-specific IgG1 titers as a function of time after Ad-lacZ gene transfer in AAV-LacZ/Ad-LacZ and Ad-LacZ only transduced mice and, for comparison, in AAV-LacZ only mice. Data are average±SD for n = 4 mice/experimental group.

Ad-LacZ transduced mice on average produced 500–700 ng/ml plasma of IgG1 anti-β-gal at days 14, 28, and 45 after gene transfer (Fig. 6). No IgG2a anti-β-gal was detected (data not shown; this is different from muscle-directed Ad-LacZ gene transfer, which results in IgG2a formation) [19]. In contrast, both AAV-LacZ only and AAV-LacZ/Ad-LacZ transduced mice failed to form antibodies against β-gal (Fig. 6).

### Inflammatory cytokine (IFN-γ) production by T cells

**Figure 7:**
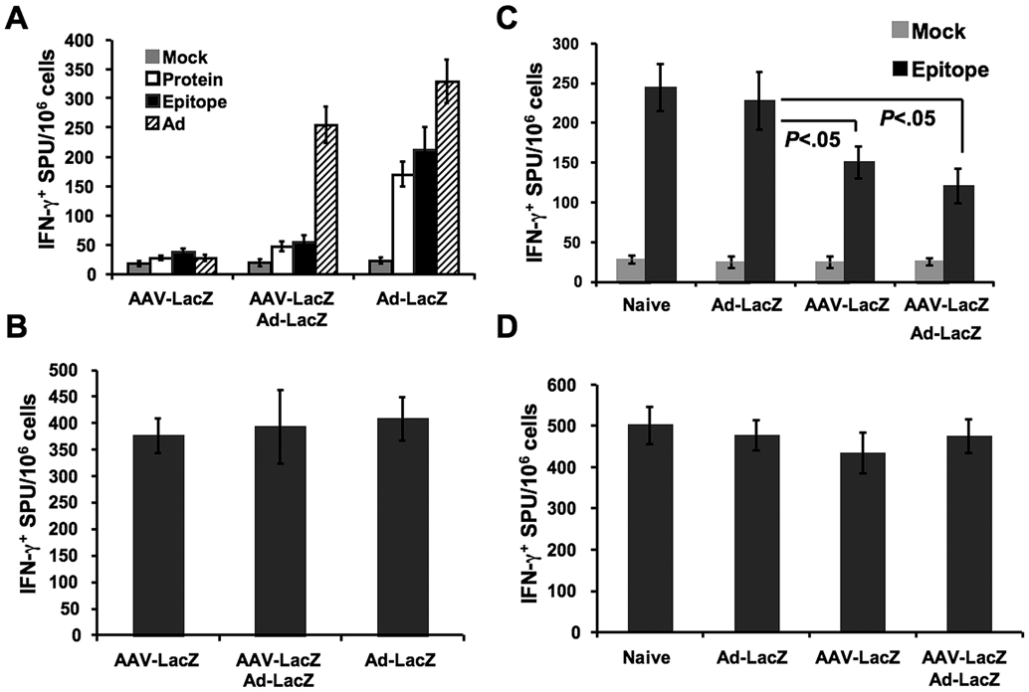
ELISpot for frequency of IFN-γ secreting lymphocytes following Ad-LacZ gene transfer. A. Frequencies in response to stimulation with mock media, β-gal protein, β-gal CD8^+^ T cell epitope, or adenoviral particles. Mice had received AAV-LacZ only (6 weeks earlier) or AAV-LacZ followed by Ad-LacZ, or Ad-LacZ only, and assayed 14 days after Ad-LacZ administration. B. Positive control stimulation using 10 µg/ml of Staphylococcal Enterotoxin B (SEB) super antigen. C. Frequencies in recipient mice of adoptively transferred CD4^+^CD25^+^ splenocytes from AAV-LacZ, AAV-LacZ/Ad-LacZ, or Ad-LacZ transduced mice. Adoptive transfer was performed 10 days after Ad-LacZ gene transfer (i.e. 6 weeks plus 10 days after AAV-LacZ gene transfer) to C57BL/6 mice. Recipient C57BL/6 mice were immunized the following day with the β-gal CD8^+^ T cell epitope and assayed 10 days later. Animals receiving cells from naïve mice were included as an additional control group. D. Positive control stimulation using 10 µg/ml of Staphylococcal Enterotoxin B (SEB) super antigen. A-D. *In vitro* stimulations were performed in triplicate for individual animals. Results are average±SD for n = 4 mice/experimental group.

In order to quantify the effect of tolerance induction on activation of pro-inflammatory subsets of T cells, ELISpot assays were performed 14 days after Ad-LacZ gene transfer to naïve or AAV-LacZ pre-treated mice. Splenocytes from individual mice were re-stimulated *in vitro* with β-gal, the immunodominant β-gal CD8^+^ T cell epitopes, or heat-inactivated adenoviral particles. Frequencies of IFN-γ secreting cells were measured for Ad-LacZ mice only and AAV-LacZ/Ad-LacZ treated mice (Fig. 7A). AAV-LacZ only mice served as an additional control. Ad-LacZ induced a robust CD8^+^ T cell response to β-gal (9-fold above the frequency in mock-stimulated cultures), which was nearly undetectable in AAV-LacZ or AAV-LacZ/Ad-LacZ transduced animals. Similar results were obtained for the IFN-γ^+^ response to entire β-gal protein antigen (Fig. 7A). Ad-lacZ only and AAV-LacZ/Ad-LacZ transduced mice showed an IFN-γ^+^ response to adenoviral particles, which was somewhat reduced in the AAV-LacZ/Ad-LacZ group (Fig. 7A). All splenocyte cultures showed equally high responses to control stimulation with super antigen (Fig. 7B).

### Induction of Treg by AAV-LacZ gene transfer

Adoptively transferred CD4^+^CD25^+^ splenocytes from AAV-LacZ and AAV-LacZ/Ad-LacZ but not Ad-LacZ transduced mice were able to significantly suppress the IFN-γ^+^ response against the dominant β-gal CD8^+^ T cell epitope when compared to non-specific Treg transferred from naïve mice (Fig. 7C). In contrast, IFN-γ^+^ responses to control stimulation with super antigen were not suppressed (Fig. 7D).

**Figure 8:**
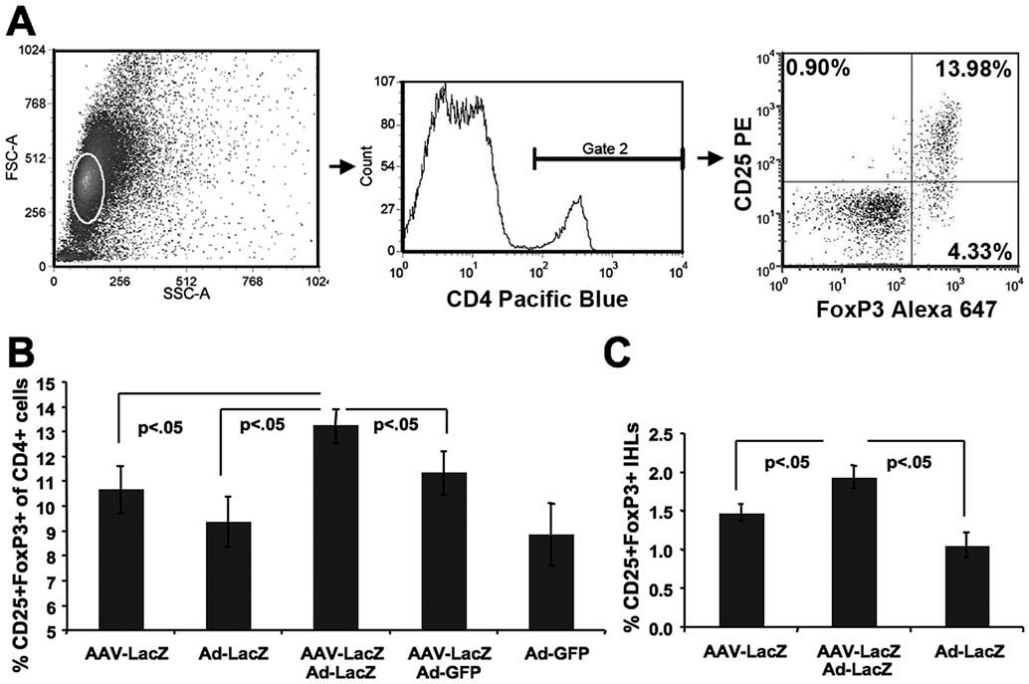
Frequencies of CD4^+^CD25^+^Foxp3^+^ Treg by flow cytometry. A. Example of analysis of splenocytes from a C57BL/6 mouse transduced with AAV-LacZ and, 6 weeks later, with Ad-LacZ. B. Frequencies of CD4^+^CD25^+^Foxp3^+^ splenocytes in AAV-LacZ, AAV2-LacZ/Ad-LacZ, AAV2-LacZ/Ad-GFP, Ad-LacZ, or Ad-GFP transduced C57BL/6 mice. C. Frequencies of CD25^+^GFP^+^ intrahepatic lymphocytes (IHL) isolated from FoxP3 reporter mice (knock in mice for FoxP3-IRES-EGFP) after transduction with AAV-LacZ, AAV-LacZ/Ad-LacZ, or Ad-LacZ. A-C. All analyses were done 10 days after Ad gene transfer. Results are average±SD for n = 4 mice/experimental group.

Furthermore, we found that the frequency of CD25^+^Foxp3^+^ Treg among CD4^+^ T cells was significantly increased in AAV-LacZ transduced mice following secondary Ad-LacZ gene transfer (Fig. 8A,B), which was not seen for secondary gene transfer with Ad-GFP control vector. Ten days after adenoviral gene transfer, the percentages of Treg were lowest in spleens of those mice that had been transduced with adenoviral vectors only, irrespective of the transgene.

Finally, we wanted to test for induction of a Treg response in the liver, which is technically more challenging. Therefore, utilized FoxP3 reporter mice (FoxP3-IRES-EGFP knock-in mice) to analyze for frequencies of Treg in form of EGFP^+^ intrahepatic lymphocytes 10 days after Ad-LacZ gene transfer. In AAV-LacZ pre-treated mice, the frequencies of Treg were significantly increased compared to AAV-LacZ transduced mice that did not receive the secondary gene transfer (Fig. 8C), while Treg were undetectable among hepatic lymphocytes from Ad-LacZ only treated mice.

## Discussion

Hepatic gene transfer with AAV vectors has been shown to induce tolerance to a number of transgene products in animal models of human disease. This treatment provided immune tolerance to the therapeutic protein and, simultaneously, therapy or, alternatively, allowed for safe administration of supplementary therapy such as enzyme replacement therapy or therapeutic gene transfer to a different organ [35]–[37]. Using this method, animals have been tolerized to F.IX, α-galacosidase, acid α-glucosidase, acid shingomyelinase, and other proteins [38]. Interestingly, these are secreted or exocytosed proteins that have been expressed in hepatocytes primarily for the purpose of systemic delivery for correction of systemic protein deficiency or cross-correction following up-take by other cell types.

### Requirements for tolerance induction

Studies with a variety of vector systems have demonstrated that requirements for tolerance induction by the hepatic route include restriction of transgene expression to hepatocytes, use of a vector with low innate immunity to reduce inflammatory signals, and lack of transgene expression in professional APCs [38]–[40]. In this context, hepatocyte-derived transgene expression leads to induction of CD4^+^CD25^+^FoxP3^+^ Treg that suppress immune responses to the transgene product [25], [26], [41]. Tolerance induction to F.IX requires Treg and likely also activation induced cell death of F.IX-specific effector T cells, which in combination may promote a shift from an effector to a regulatory response [18], [25]. Although AAV-2 vectors transduce only a small portion of hepatocytes (typically ∼5%), robust transgene expression can induce tolerance. In the case of F.IX, we estimated that a systemic level of 30–50 ng/ml plasma is required for tolerance induction [18]. It is therefore conceivable that adequate secretion of a protein from few hepatocytes results in delivery to and tolerogenic antigen presentation by professional APCs, which may even be located in lymphoid organs distal from the liver. Certainly, the spleen is a site of processing of systemically circulating antigens. However, our new data demonstrate that induction of immune tolerance by AAV gene transfer does not require systemic expression. Expression of a cytoplasmic protein in relatively few hepatocytes (1–3%) was sufficient to completely protect from antibody formation, to nearly eliminate activation of transgene product-specific CD8^+^ T cells, and to substantially reduce hepatotoxicity and T cell infiltrates following insult with a first-generation Ad vector.

### Implications for antigen presentation and T cell activations

In hepatic AAV-LacZ gene transfer, the expressed β-gal antigen is not ignored but rather presented to T cells in a fashion that results in Treg induction. In our previous studies with F.IX, we found that splenic CD4^+^ T cells from AAV-F.IX hepatic transduced mice were capable of suppressing the cellular immune response to Ad-F.IX gene transfer following adoptive transfer [22]. Based on phenotyping of splenocytes of donor mice, we hypothesized that CD4^+^CD25^+^FoxP3^+^ Treg mediated suppression. Here, we demonstrated more directly that splenic CD4^+^CD25^+^ Treg of tolerized mice are able to suppress the transgene product-specific CD8^+^ T cell response. Moreover, we found that secondary gene transfer with an Ad vector expressing the same transgene causes an increase in FoxP3^+^ Treg frequency in liver and spleen. This amplification of the regulatory response likely controlled inflammation and liver damage, resulting in a 10-fold reduction of CD8^+^ T cell infiltrate in the liver despite the presence of immunogenic adenoviral antigens. This suggests that antigen expressed in the cytoplasm of a small portion of hepatocytes can be effectively presented in a tolerogenic fashion, thereby directing a Treg response that actively down-regulates inflammatory T cells. Increase of Treg frequency and induction of suppression upon Ad gene transfer was antigen-specific, i.e. linked to the expression of the same β-gal antigen by the tolerizing and the challenging vector. If the Ad encoded transgene was switched to GFP, liver damage was identical to that induced by Ad-LacZ gene transfer to naïve mice. The APC that is inducing the suppressive T cell population remains to be identified. Direct presentation of antigen by hepatocytes to T cells and indirect pathways utilizing bone marrow-derived APCs or endothelial cells have been proposed [42], [43].

### Implications for gene therapy

Even if the T cell response against Ad gene transfer was completely suppressed *in vivo*, this vector still causes inflammation and hepatotoxicity via a non-antigen-specific innate response [44], [45]. Despite these inflammatory signals, the destructive response against Ad-LacZ transduced liver was suppressed enough to achieve a 5-fold increase in the number of β-gal expressing hepatocytes compared to AAV-LacZ only transduced livers (when analyzed 1.5 months after secondary gene transfer, a time point when expression from the Ad-LacZ vector had been completely eliminated in control mice). In prior experiments with a F.IX transgene, we found that secondary Ad-F.IX gene transfer to livers of AAV-F.IX transduced mice increased the numbers of F.IX expressing to >10% of hepatocytes at later time points [22]. This may reflect higher expression levels of F.IX compared to β-gal upon hepatic AAV-2 gene transfer, differences in the characteristics of the transgene products, or differences between strains of mice. Nonetheless, our new data show that systemic expression is not a requirement for tolerance induction, albeit we cannot rule out that systemic delivery further enhances tolerance. It will be of interest to investigate whether expression of cytoplasmic or secreted proteins may alter the relative contributions of Treg induction and other tolerance mechanisms (such as T cell deletion or anergy) to induction of unresponsiveness of the transgene product-specific T cell population [46].

Recently, we found that hepatic AAV-F.IX gene transfer results in tolerance induction to F.IX in multiple strains of mice over a wide range of vector doses using a serotype 8 vector [47]. Combined, these observations are encouraging for development of a more universal, hepatic gene transfer-based tolerance protocol that is applicable to antigens with different cellular localizations and less influenced by genetic factors inherent to the recipient of gene transfer. Future protocols could incorporate secondary gene transfer (following tolerance induction) using more effective vectors that may otherwise be too immunogenic. Moreover, improved AAV vectors may transduce a higher number of hepatocytes, which has the potential to improve therapy and tolerance induction [47].
